# Limited ecosystems research as a persistent gap in One Health frameworks for emerging infectious diseases

**DOI:** 10.1093/biosci/biag111

**Published:** 2026-07-22

**Authors:** Nadja Kabisch, Jan Felix Drexler

**Affiliations:** Leibniz University Hannover, Institute of Earth System Sciences, Physical Geography and Landscape Ecology Section, Schneiderberg 50, 30167 Hannover, Germany; Charité–Universitätsmedizin Berlin, Corporate Member of Freie Universität Berlin and Humboldt-Universität zu Berlin, Institute of Virology, Charitéplatz 1, 10117 Berlin, Germany; German Centre for Infection Research (DZIF), associated partner site Charité, Berlin, Germany

**Keywords:** ecosystem degradation, research funding, landuse and landcover changes, climate change, zoonosis, pandemic

Decades ago, the One Health framework emerged as an interdisciplinary public health approach addressing the interconnected health of humans, other animals, and the wider environment (FAO et al. 2008, OHHLEP et al. 2022). The One Health framework is frequently used to describe emergence of new pathogens, particularly in humans. Technological advances fueling pathogen discovery including unbiased sequencing approaches, AI-supported analytical methods, and expanding global surveillance efforts have accelerated the discovery of previously unrecognized pathogens in wildlife and other animal hosts (Carlson et al. 2025). However, pathogen discovery alone provides limited insight into the ecological, ecosystem related mechanisms governing pathogen maintenance, amplification in reservoirs, and zoonotic spillover.

In this Viewpoint, we use the term *ecosystem* to refer to the dynamic interactions among organisms, including pathogenic microorganisms such as viruses and bacteria, their hosts’ habitats, predator-prey relationships, landuse and landcover changes with anthropogenic impacts across spatial and temporal scales and climatic conditions. Although environmental variables are increasingly incorporated into One Health research, efforts to investigate ecosystem-related ecological processes remain comparatively underrepresented within emerging infectious disease research (Zinsstag et al. 2011). Existing studies often focus on specific components of ecosystem change, such as antimicrobial resistance (Essack 2018), vector ecology (Arisco et al. 2024), or wildlife-associated virus prevalence (Eby et al. 2023). We argue that there is a need to move beyond documenting pathogen occurrence in a siloed setting toward understanding how ecosystem processes, structure, and function and also human social and behavioral processes shape pathogen transmission dynamics and zoonotic risk in an integrated approach, ideally within long-term studies and ecologically validated replicates.

We suspect, however, that financial, methodological, and structural barriers continue to limit ecosystem-oriented research relative to pathogen discovery efforts that are methodologically less demanding and often lack broader ecological context. In this Viewpoint, we describe below several challenges as illustrated in figure 1 and summarized in box 1 to comprehensive studies based on our own and other researchers’ experience and suggest means to overcome these challenges.

- **Species-specific sampling complexity**
  Sampling designs need to fit the animal species under study, including vertebrate and invertebrate organisms with differing home ranges and responses to anthropogenic landcover change. Capturing ecosystem-level dynamics across multiple taxa with sufficient ecological replicates and sampling plots requires exhaustive assessments that are methodologically cumbersome (Brouard et al. 2015), potentially dangerous particularly in tropical settings, and resource-intensive. At the same time, minimum methodological standards allowing comparisons across studies remain insufficiently harmonized (Schwantes et al. 2025).
- **Lack of ecologically validated replicate data**
  Longitudinal studies across ecologically validated replicates are needed to account for seasonal and climatic variation affecting both environmental conditions and pathogen circulation in reservoir hosts (Eby et al. 2023). However, such datasets remain limited, constraining efforts to understand pathogen emergence across space and time. Historical ecosystem-level data are frequently unavailable at local scales, including information on past pollution affecting biodiversity and trophic interactions.
- **Limitations in resolution and availability of historical spatial data**
  Remote sensing has substantially improved ecosystem monitoring capacities, and research linking landuse and landcover change with infectious disease emergence has expanded substantially in recent years. Although availability of high-resolution data from commercial sensor systems has increased, spatial resolution of publicly available sensor data is often not sufficient to assess plot-level dynamics (e.g., with Landsat data available since the late 1970s with a resolution of only 100 m and later 30 m), and long-term coverage is scarce (Zeng et al. 2022). Remote sensing can therefore complement but not replace ground-level in-situ ecological assessments.
- **Complexity of ecosystem-scale interactions**
  Ecosystems overlap entailing interactions between wildlife, domestic animals, and livestock along rural-to-urban gradients. Such interactions can lead to pathogen exchange and amplifications. Ecosystem-based interactions, but also alterations do not affect all hosts, vectors, or pathogens uniformly. Species differ in ecological specialization, mobility, trophic interactions, and tolerance to anthropogenic disturbance such as landuse changes and climate change, producing highly context-dependent pathogen dynamics across disturbance gradients (Ecke et al. 2025). Ecosystem restoration as a transient anthropogenic disturbance (ibid) could be framed as a nature-based solution to reducing risks of infectious diseases when also benefiting biodiversity and providing ecosystem services but evaluating these effects and outcomes is challenging. In addition, certain host taxa and associated pathogens may only occur at the extremes of disturbance gradients, limiting robust assessments of the extent of ecosystem alteration on pathogen abundance and genomic diversity (Hermanns et al. 2023).
- **Microbial interaction complexity**
  Understanding ecosystem interactions among micro-organisms remains particularly challenging because interactions within and between pathogen taxa are still poorly resolved. For example, insect-specific viruses may differentially affect arbovirus infectivity (Jansen et al. 2025), but many additional interactions likely remain undiscovered. Experimental validation of such interactions is technically difficult and resource-intensive. Similarly, pathogens vary widely in environmental stability, replication intensity, and shedding routes, leading to different concentrations in given analyzed biological specimens. Arguably, pathogens with different levels of host specificity, environmental stability, and shedding patterns will respond differently to ecosystem alterations in a context-specific manner and in parallel show stark differences in laboratory detectability that need to be accounted for.
- **Constraints of serological and molecular surveillance**
  Serological approaches can complement molecular detection by assessing lifetime exposure to pathogens across host populations and environmental gradients. However, antibody assays for emerging pathogens often lack validation across wildlife species and remain difficult to standardize, many being developed and validated for one host species only. Similarly, antibody tests can yield positive results with antigens derived from different pathogens, and those cross-reactive immune responses must not be interpreted as direct evidence for a specific pathogen having infected the vertebrate under study (Fischer et al. 2021). At the same time, direct pathogen detection commonly done by molecular tools can vary with seasonality, infection dynamics, host immune status and other traits and again, sampling in few locations and time points may bias test results substantially (Chen, 2023).
- **Complexity of climate change-driven ecological alterations**
  Climate-driven ecosystem changes can alter habitat suitability and vector abundance exemplified by increasing suitability of previously temperate regions in Europe for tropical vectors such as *Aedes albopictus* and related pathogen emergence including dengue and chikungunya viruses (Farooq et al. 2025). Understanding these ecosystem changes and related processes requires integrating observational studies on vector ecology with long-term and short-term meteorological, landuse, and landcover variables. Generating such high-quality datasets across temporal, local and regional scales remains challenging and resource-intensive.
- **Challenge of integrating social and ecological systems research**
  Understanding relationships between climate, landuse, landcover, human behavior, and disease occurrence requires interdisciplinary collaboration between natural sciences, life sciences and particularly the social sciences. In addition, conducting community-based research, particularly involving local communities and indigenous people in inter- and transdisciplinary approaches, is methodologically sensitive, time-consuming, and resource-intensive (Jeleff et al. 2022), but essential for understanding local socio-ecological systems. Conceptual understandings, use of scientific language and terms can also differ by disciplines in social, natural, and life sciences and may challenge truly interdisciplinary research.
- **Ethical and regulatory complexity**
  Ethical approvals and equitable access-and-benefit-sharing frameworks under national and international legislation, including the Nagoya Protocol (Colella et al. 2023), are essential components of ecosystem-scale research. However, coordinating permissions involving humans, wildlife, pathogens, and international sample exchange often requires substantial administrative effort, time, and financial resources.

Box 1 The One Health framework enhances the understanding of the emergence of infectious diseases by considering the interconnected health of humans, animals, and the environment. However, the use of an ecosystem lens to understand the ecology of macro- and microorganisms’ underlying pathogen maintenance in reservoirs and spillover to other hosts including humans remains limited. Addressing this gap requires long-term interdisciplinary research within frameworks focused on ecosystems, supported by long-term funding. Several challenges hinder broader application of an ecosystem lens in research on the ground, including i) Species-specific sampling complexity; ii) Lack of longitudinal ecosystem datasets; iii) Limitations in spatial data resolution; iv) Complexity of ecosystem-scale interactions; v) Microbial interaction complexity; vi) Constraints on serological and molecular surveillance; vii) Climate-driven ecological change altering habitats and vectors; viii) Challenging integration of social science based research; ix) Ethical and regulatory complexities and financial burdens to international research. Ignorance of the ecosystem scale in pathogen dynamics will hinder pandemic preparedness and ultimately cause larger societal and economic costs than adequate funding and research designs.

We argue that a strong commitment by international and national research funders to long-term interdisciplinary research integrating natural, social, and life sciences within ecosystem focused and ecologically valid study frameworks would be essential to overcome the outlined challenges. Such support would facilitate the generation of comparable ecosystem-scale evidence across studies and improve our ability to derive robust conclusions relevant for pandemic preparedness and the well-being of humans, other animals, and the environment as originally envisioned within the One Health framework. Robust conclusions and resulting evidence-based recommendations regarding the links between ecosystem change, climate change, human behavior, and pathogen emergence are also essential to prevent misinterpretation, science scepticism, and conspiracy claims, as observed in other environmental and public health contexts (Kabisch and Egerer 2025). Broad international funding initiatives for pandemic preparedness are feasible, as demonstrated by CEPI, the Coalition for Epidemic Preparedness Innovations, focusing on vaccine development, and the NIH-funded Centers for Research in Emerging Infectious Diseases (CREID) Network, which conceptually incorporated ecosystem components before being discontinued in 2025 according to revised national funding priorities (Kaiser 2025). Despite increasing rhetorical recognition of environmental dimensions within One Health, we conclude that ecosystem-focussed research remains insufficiently integrated into emerging infectious disease research and funding structures. This imbalance is counterproductive for efforts seeking to understand pathogen emergence and prepare for future pandemics.

**Figure 1.**
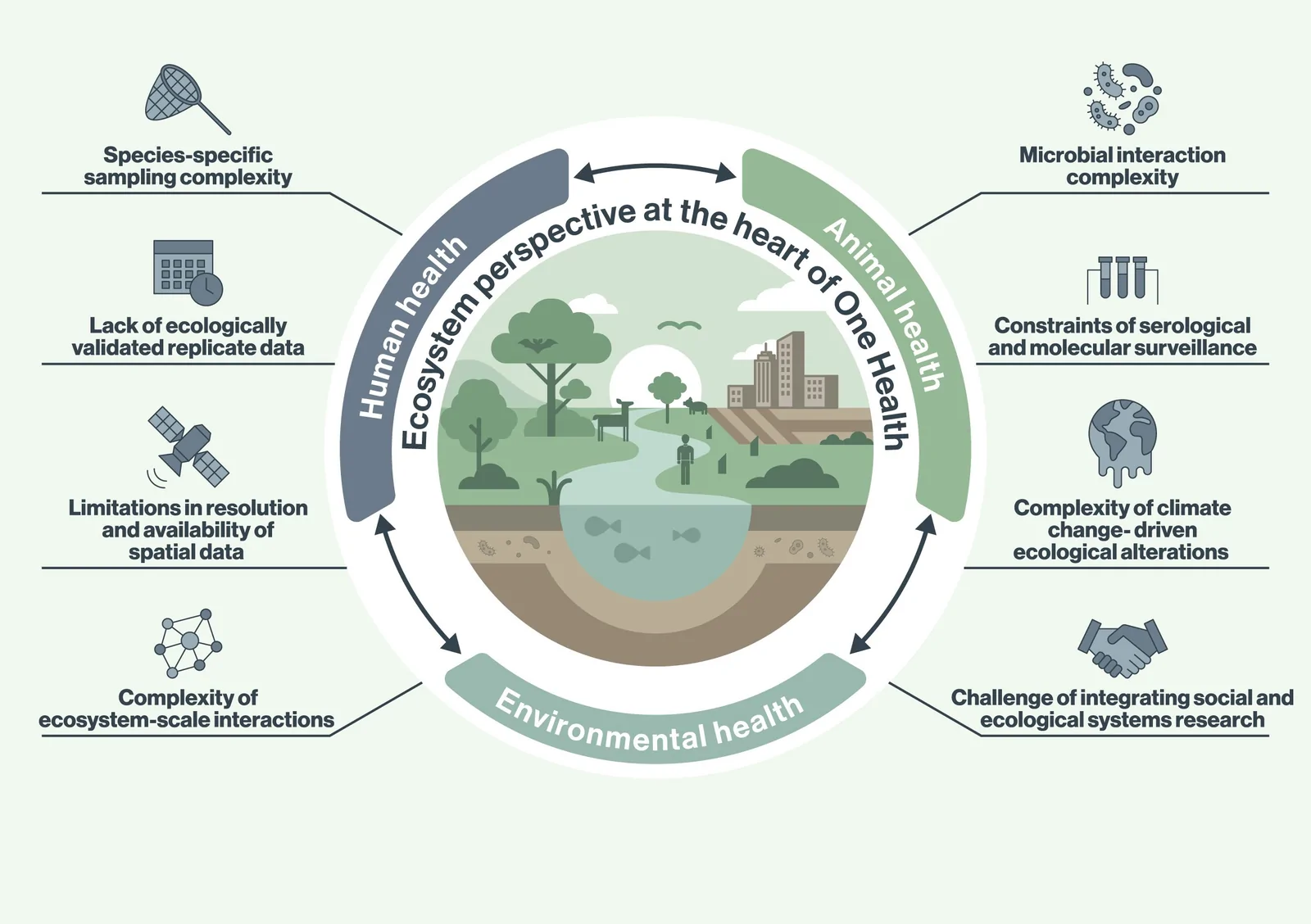
Challenges limiting an ecosystem perspective at the heart of One Health. (Note: The figure was conceptualized by the authors. The used figure elements (graphics/icons) were custom drawn by Imre Sebestyén (unitgraphics.com).)

## Data Availability

No new data were generated or analysed in support of this research.

## Author Biography

Prof. Nadja Kabisch is a professor for Digital Landscape Ecology at the Leibniz Universität Hannover, where she focuses on urban environmental sciences and ecosystem services. Her work primarily explores how urban nature contributes to biodiversity, climate resilience, and human health and well-being. Prof. Kabisch is recognized for her interdisciplinary work, integrating ecological, remote-sensing, social, health and planning perspectives to enhance urban sustainability and livability.

Prof. Jan Felix Drexler is a professor for Virus Epidemiology at the Charité-Universitätsmedizin Berlin, known for his research on emerging viruses and their transmission, focusing on how these pathogens can shift from animal reservoirs to humans and spread between humans. His work has significant implications for public health, especially in developing strategies to prevent and control outbreaks caused by emerging viruses.
